# Hyaluronic Acid: A Valid Therapeutic Option for Early Management of Genitourinary Syndrome of Menopause in Cancer Survivors?

**DOI:** 10.3390/healthcare10081528

**Published:** 2022-08-13

**Authors:** Rossella E. Nappi, Silvia Martella, Francesca Albani, Chiara Cassani, Ellis Martini, Fabio Landoni

**Affiliations:** 1 Department of Clinical, Surgical, Diagnostic and Pediatric Sciences, University of Pavia, 27100 Pavia, Italy; 2 Unit of Reproductive Medicine, Gynecological Endocrinology and Menopause, IRCCS S. Matteo Foundation, 27100 Pavia, Italy; 3 Unit of Preventive Gynecology, IRCCS European Institute of Oncology, 20141 Milan, Italy; 4 Gynecological Endocrinology Clinic, Unit of Internal Medicine and Endocrinology, IRCCS Maugeri, 27100 Pavia, Italy; 5 Unit of Obstetrics and Gynecology, IRCCS S. Matteo Foundation, 27100 Pavia, Italy; 6 Gynecologic Oncology Unit, Department of Obstetrics and Gynecology, ASST-Monza, San Gerardo Hospital, University of Milano-Bicocca, 20900 Monza, Italy

**Keywords:** vulvar and vaginal atrophy, genitourinary syndrome, menopause, hormone-dependent cancer, hyaluronic acid

## Abstract

Genitourinary syndrome of menopause (GSM) is a chronic condition affecting a large number of women, with a major impact on their urogenital health and sexual function. It occurs at midlife because estrogen levels decline with menopause enhancing aging-related changes of the functional anatomy of the urogenital system. Unfortunately, GSM may occur early in the lifespan of women or be exacerbated following anticancer treatments, such as chemotherapy, ionizing radiation, or surgical removal of reproductive organs. Symptoms of GSM are often under-reported by women, under-estimated and under-diagnosed by health care providers (HCPs), and subsequently under-treated, despite their profound negative impact on the quality of life. The mainstay of vaginal treatments is local estrogen therapy (LET) ensuring an effective management of moderate to severe symptomatic GSM. However, LET is generally contraindicated in women with a history of hormone receptor positive cancer, due to the fear of increased recurrence or possible interference with endocrine adjuvant therapies. Among non-hormonal treatments, hyaluronic acid-based moisturizers have shown promising clinical results both in healthy women and in cancer patients or survivors. Its strong water-binding properties provide lubricating and moisturizing effects, which contribute to maintaining a proper level of hydration and viscoelasticity in several body parts, including the urinary tract and genital tissues. Hyaluronic acid-based moisturizers are effective, safe, and well tolerated; therefore, they may represent a valid option for the early management of GSM-associated symptoms in every woman with a history of cancer who is unable or unwilling to undergo hormone-based therapies. Hence, the aim of this review was to provide an overview of GSM etiology and treatment in women with natural or iatrogenic menopause, with a focus on the use of hyaluronic acid as a prophylactic treatment in the context of an integrated management protocol for cancer patients.

## 1. Introduction

Genitourinary Syndrome of Menopause (GSM), previously known as vulvovaginal atrophy (VVA), atrophic vaginitis, or urogenital atrophy, is a new, more all-encompassing, and accurate term including the variety of genital, sexual, and urinary symptoms associated with the anatomical and functional changes in vulvovaginal tissues and the pelvic area that occur during menopause and aging [1]. In European countries, the mean age of menopause is between 46.7 and 50.1 years; considering the increase in life expectancy, this means that the postmenopausal period corresponds to approximately 40% of a woman’s life [2]. VVA is a component of GSM [3] and can arise at any age but is particularly common in the post-menopausal period because it results from decreased levels of estrogen in urogenital tissues [4]. VVA is characterized by vaginal dryness, burning, irritation, and light bleeding after intercourse [4], and its complications may include dyspareunia, dysuria, and recurrent urinary tract infections (UTIs) [5].

GSM is a chronic condition negatively affecting women’s quality of life (QoL), not only from a physical point of view, but also from a psycho-relational one. Indeed, GSM has a strong adverse impact on women’s intimate and sexual health, and on their self-esteem [6]. It can also alter sleep, temperament, work, and leisure activities, and reduce the zest for life [6]. At midlife, symptoms related to GSM are often overlooked, with more attention paid to the long-term consequences of hormonal changes of menopause for bone mass and the cardiovascular system. Moreover, hot flushes and night sweats are considered the typical menopausal symptoms, whereas those associated with GSM are often related to aging itself [6]. Therefore, women are rarely asked specific questions about their urogenital health and both misinformation and reluctance to address genital and sexual problems lead GSM to be often under-diagnosed and under-treated in routine consultations [7]. 

The situation is particularly problematic in the case of cancer patients; in fact, the same profound changes experienced by women because of natural menopause may occur in cancer survivors. Several oncologic treatments induce estrogen deficiency, which results in temporary or permanent amenorrhea [8,9]. This is the case with surgical removal of reproductive organs, endocrine and non-endocrine chemotherapy, and radiotherapy. Advances in cancer control strategies have greatly increased the chances of survival; as a result, a growing number of women must deal with the physical, psychological, and relational consequences of premature iatrogenic menopause [10]. Among these, GSM represents one of the adverse events that severely affect QoL. In cancer patients and/or survivors, GSM management is even more difficult than in women experiencing physiological menopause [10,11]. Indeed, there is a lack of evidence about the safety of available local hormonally based therapies in the oncological setting [10,11]. That being so, women with or at high risk of cancer have the additional burden of a very restricted therapeutic choice for handling GSM symptoms. This makes the improvement of QoL through GSM care enhancement a challenging issue.

Hyaluronic acid (HA), a non-sulfated glycosaminoglycan, is an essential component of connective, epithelial, and neural tissues [12]. Its strong water-binding properties provide both lubricating and moisturizing effects, which contribute to maintaining a proper level of hydration and viscoelasticity in several body parts, including the urinary tract and genital tissues [13]. Endocrine changes associated with menopause bring about several consequences for urogenital tissues, including reduced synthesis of HA and mucopolysaccharides in the extracellular matrix (ECM) [14]. HA-based moisturizers have shown promising clinical results in the treatment of GSM-associated symptoms, both in terms of efficacy, safety, and tolerability [15]. 

This narrative review aims at providing an overview of GSM etiology and treatment in women with natural or iatrogenic menopause, focusing on the use of HA as a prophylactic treatment in the context of an integrated management protocol for cancer patients.

## 2. Search Strategy

We carried out the present review by analyzing different electronic databases, such as MEDLINE (PubMed), Cochrane Central Register of Controlled Trials, and Scopus. Eligible articles were searched by using the following keywords and MeSH terms, or combinations of them: “vulvo vaginal atrophy”, “genitourinary syndrome of menopause”, “estrogens”, “hyaluronic acid”, “hormone-dependent tumors”, and “hormone replacement therapy”. 

The selection was performed by screening titles and abstracts of articles found through the electronic searches (over 2000 between 2002 and 2022). The full text of all relevant papers was evaluated for inclusion (103 articles), as well as other relevant cited articles when appropriate. 

## 3. Etiology of GSM

GSM is mainly the consequence of a decrease in the circulating level of estrogen, a crucial regulator of urogenital physiology [5]. The effects of estrogens are mediated by binding to specific receptors, estrogen receptor α (ER-α) and β (ER-β), and nuclear transcription factors that are highly expressed in the female genital and lower urinary tracts [6,16]. Before menopause, estrogens are essential in maintaining thickness and moisture of the vaginal epithelium, and in ensuring a sustained blood flow. Androgens, directly or being converted locally into the tissues, may also play a role [14]. In a healthy condition, the vaginal wall can exfoliate and release high amounts of glycogen into the vaginal lumen. Glycogen is then converted into lactic acid by *Lactobacilli*, commensal microorganisms dominating the normal vaginal microbiota, thus keeping the pH in an optimal acidic range (3.5–4.5) [17]. The decline in estrogen levels associated with menopause triggers several transformations of the genital tissues, which include thinning of the vaginal epithelium, connective tissue proliferation, decreased vaginal blood flow, and loss of tissue elasticity due to elastin fragmentation and collagen fibers hyalinization [18,19]. A thinner vaginal epithelium is associated with decreased exfoliation of glycogen-rich epithelial cells, which in turn causes an increase in vaginal pH (between 5.0 and 7.5), allowing the growth of pathogenic bacterial flora [5,19,20]. This altered microbiota may predispose to develop vaginitis and other urogenital infections [21] along with the most typical symptoms of vaginal dryness and pain. Estrogen deficiency induces similar atrophic changes also in the urinary tract, where estrogen receptors are abundantly expressed [16,22]. Consequently, women with GSM often suffer from urinary symptoms such as recurrent infections, incontinence, urgency, and dysuria [5,17,23]. Other anatomical and physiological changes in urogenital tissues include that the vagina may shrink, shorten, and lose its elasticity and flexibility. The labia minora thin and regress, while the labia majora lose their subcutaneous fat. The introitus may contract, and pubic hair may become scarce [5,24,25]. Blood flow and secretions decrease, resulting in a pale, dry vaginal epithelium that may be associated with petechiae [5,17,24,26]. Even the urethra may lose thickness and elasticity; the contractility of the urinary sphincter, as well as the strength and control functionality of pelvic floor muscles, may decrease. Finally, the capacity, contractile ability, and compliance of the bladder decline [5,24,25].

Such macroscopic changes are the result of profound modifications occurring at tissue and cellular level. Apart from the lack of proliferation and stratification of vaginal and urethral epithelial cells [24,25], hypoestrogenism strongly influences ECM remodeling, causing alterations in collagen and elastin fibers, and a depletion of glycosaminoglycans (GAGs) [14]. Among the latter, HA is particularly affected by estrogen deprivation; indeed, production of HA is significantly increased (up to 11-fold) by estradiol [27] and progressively declines both with aging and the onset of natural or iatrogenic menopause. Overall, these effects result in a loss of strength, elasticity, and moisture at the urogenital dermal layer [4,5,15,25].

## 4. GSM in Cancer Patients

Women diagnosed with cancer in the premenopausal period may experience temporary or permanent amenorrhea and develop GSM symptoms [28,29]. Ovarian dysfunction or failure is an adverse event associated with several anticancer treatments [30,31]. Ionizing radiation also has a detrimental effect on gonadal function. The ovaries are particularly exposed during pelvic radiotherapy of gynecological, urological, and gastrointestinal cancers. Indeed, this treatment is significantly associated with vaginal morbidity [32]. Several studies report that a high percentage of women surviving to cervical cancer, a tumor often diagnosed at a relatively young age, suffer from menopausal and urinary symptoms, as well as sexual dysfunction [33,34,35]. Premature menopause can also be the result of surgical removal of reproductive organs. Women carrying germline mutations in BRCA1 or BRCA2 tumor suppressor genes are at high risk of developing breast and ovarian cancer, and such risk increases with age [36]. Prophylactic bilateral salpingo-oophorectomy has proven to be more effective than surveillance/screening and chemoprevention in reducing cancer mortality. As an adverse event, iatrogenic menopause resulting from risk-reducing surgery seems to be related to more severe symptoms such as hot flushes, sleep disturbances, vaginal dryness, and sexual dysfunction compared to natural menopause [36,37]. 

Breast cancer (BC) remains a major health concern among women [11,38,39]. Although the incidence rate of BC is rising, the mortality rate is decreasing thanks to widespread early screening and advanced medical therapies [40,41]. As BC survivors increase, long-term adverse events, including symptoms related to iatrogenic menopause, are becoming more and more important [42]. The average age of BC onset in Western countries is 62 years; therefore, most women are already in menopause when diagnosed. Nevertheless, approximately 25% of all new cases occur in premenopausal women [43]. Besides non-hormonal chemotherapy, which is known to induce amenorrhea [42], hormonal therapy is widely used for BC treatment [44]. The two major classes of anti-estrogen drugs are Selective Estrogen Receptor Modulators (SERMs) and Aromatase Inhibitors (AIs) [41]. Tamoxifen, a SERM that acts by blocking ER binding, is the most widely used drug for the treatment of hormone-dependent BC [45]. The prevalence of GSM associated symptoms in women under treatment for BC or with an history of the disease is estimated to be higher than in the general female population being over 70% compared to over 50% in the general population of postmenopausal women, respectively [46,47,48]. The development of aging-associated changes in the urogenital tissues, including the reduction of intercellular mucopolysaccharides and hyaluronic acid in the dermal layer [6], may be accelerated by AIs and SERMs. After 1 year, young women undergoing adjuvant BC therapy (non-endocrine and endocrine) experience a heavy impairment in significant domains of QoL, including sexuality [49]. AIs seem to be associated with increased GSM symptoms compared to tamoxifen [50,51,52,53,54], probably because of the pro-estrogenic effect of the latter on the vulvovaginal epithelium [55]. Indeed, BC patients treated with AIs experienced GSM with significantly higher rates of vaginal dryness (16.3%) and dyspareunia (17.8%) compared to women taking tamoxifen (8.4% and 7.5%, respectively) [39]. As the use of AIs is raising, the absolute number of BC patients with symptomatic atrophic vaginitis is also likely to increase [56].

In women developing menopausal symptoms because of cancer treatments, GSM has a detrimental effect on QoL [9,47]. A survey revealed that oncologists are aware that GSM is a major problem for BC survivors, strongly influencing women’s sexual health and increasing the probability of urinary tract infections. However, less than half of oncologists directly inform patients that GSM is one of the possible consequences of adjuvant treatments. In most cases, GSM is discussed at the follow-up visit only if the patient complains of symptoms [47]. This occurs in spite of the evidence that GSM is an epidemic condition in postmenopausal women with BC, with a highly significant burden of disease [57]. It is often under-reported by women, who are reluctant to talk about genital and sexual symptoms; under-estimated and under-diagnosed by HCPs, who most often consider it as a simple sexual or age-related discomfort; and consequently, under-treated [4]. The Women’s EMPOWER survey showed that many times women did not reveal their symptoms with their HCPs because of embarrassment, but most of them would like to try a product for symptom relief [58,59].

It would be helpful if patient education in the management of urogenital health could be routinely incorporated into the care of cancer survivors [9], given the evidence that GSM signs or symptoms are likely to appear [26]. Moreover, physical examination, including evidence of GSM, should be part of routine care of cancer women with sexual concerns in the oncology setting [60].

## 5. GSM Management

Unlike vasomotor symptoms (such as hot flushes, night sweats, and heart palpitations), which usually improve over time even without treatment, GSM symptoms can progressively worsen and will not resolve without targeted intervention [24,61]. First-line therapies for GSM include non-hormonal, long-acting vaginal moisturizers and low-dose vaginal estrogens [61,62,63,64]. According to the last position statement of North American Menopause Society [61] and expert reviews [11,65,66], non-hormonal treatments should be considered the first line therapy for patients with BC or hormone-dependent tumors. In addition to the use of vaginal moisturizers and lubricants, regular use of vaginal dilators as well as pelvic floor therapy has been recommended for symptomatic vaginal atrophy to reduce pain with vaginal penetration [11].

Additional pharmacological therapies include intravaginal dehydroepiandrosterone (DHEA) ovules and ospemifene. DHEA, an inactive precursor of sex steroids, is converted locally into androgens and/or estrogens. It has been shown to be effective in improving several GSM symptoms with a high profile of tolerability [67]. Data of local DHEA in cancer survivors are limited and therefore its use is still debated for this category of patients [11,61]. Ospemifene is a systemically administered SERM that has been proven to effectively improve vaginal dryness and dyspareunia [68,69]. It is indicated for the treatment of GSM moderate to severe associated symptoms in healthy post-menopausal women [70]. Since its antiestrogenic or neutral effects on the breast tissue, ospemifene might be an option for BC survivors who completed endocrine adjuvant therapy but is contraindicated for patients still on treatment [23,70,71]. This implies that, during the 5–10 years of adjuvant therapy, patients with BC or hormone-dependent tumors can safely resort to non-hormonal treatments only [55].

Non-hormonal treatments for GSM include vitamin D/E vaginal suppositories and an oil-in water emulsion for vaginal application [72,73]. However, only limited data are available and further evidence is needed to support the efficacy of these products [48]. Women with pain isolated at the vulvar vestibule can feel relief in using topical lidocaine [74]. Additional non-hormonal treatment options that may be particularly suitable for BC patients with GSM symptoms include laser therapy or other thermal energies [75,76]. They are thought to induce vaginal tissues remodeling by promoting collagen synthesis, thus increasing elasticity and integrity of the vaginal epithelium [11,61,75,76]. Several small scale, non-randomized clinical studies have shown vaginal health improvement in postmenopausal women undergoing laser therapy, but many open questions remain, for instance, which of the two existing technologies (the micro-ablative carbon dioxide laser and the non-ablative vaginal erbium YAG laser) among others, are the most appropriate for GSM [48,75]. Interestingly, a retrospective study showed that fractional microablative CO_2_ laser therapy was effective in reducing GSM symptoms in BC survivors [77]. These findings have been recently confirmed in a prospective cohort study using fractional microablative CO_2_ laser in women with a history of BC, irrespective of being previously or currently on endocrine therapies [78]. Nevertheless, the application of laser therapy appears to be limited in clinical practice due to its high cost [79]. Moreover, a recent randomized clinical trial demonstrates that the evidence for vaginal laser is not clear-cut against placebo [80]. Even though laser therapy is emerging as an alternative treatment for BC patients with GSM, there is little evidence available as to its value in this setting. As a result, several international scientific communities have released consensus statements, with the majority reporting that the routine use of laser should not be recommended. 

Depending on the severity and persistence of symptoms, a treatment with low dose LET may be chosen only after careful consultation between the woman’s gynecologist and oncologist to evaluate potential risks [11,66]. Nevertheless, current clinical data are insufficient to confirm the safety of low-dose vaginal estrogens in these patients [11,61].

Finally, yet importantly among non-hormonal therapeutic agents, HA has some distinctive features that make it a potential good candidate for the management of GSM symptoms in the oncological setting.

## 6. Hyaluronic Acid: A Multifaceted Molecule

HA belongs to the family of GAGs. It has a simple chemical structure and differs from other GAGs in the absence of sulphate groups or covalently bound proteins. It is a large, unbranched polymer composed of repeated disaccharide units of glucuronic acid and N-acetylglucosamine [12,81]. In physiological conditions, it consists of 2000–25,000 disaccharides with corresponding molecular weights (MW) in the range of 10^6^–10^8^ Da. HA has notable hydrodynamic characteristics, especially in terms of viscosity and water-retaining capacity. The polymer has both charged and hydrophobic faces, due to the carboxyl groups of glucuronic acid and the disaccharide component, respectively. In dilute solutions, the domain occupied by each HA molecule expands due to mutual repulsion between the carboxyl groups, thus occupying a large volume, with water trapped within the structure. When solutions are more concentrated, molecules of HA become intertwined and form a continuous but porous meshwork: this matrix exerts the so-called “swelling pressure” that contributes to conferring to HA its peculiar viscoelasticity [82]. Furthermore, HA solutions are non-thixotropic: they are able to recover their original structure and viscosity proceeding through the same intermediate states of the degradation process, so the breakage of the polymeric network is transient and reversible [12,81]. These properties provide resilience and malleability to many tissues. HA also forms a multivalent template for interactions with proteoglycans and other extracellular macromolecules that is important in the assembly of extracellular and pericellular matrices [82,83].

**Figure 1:**
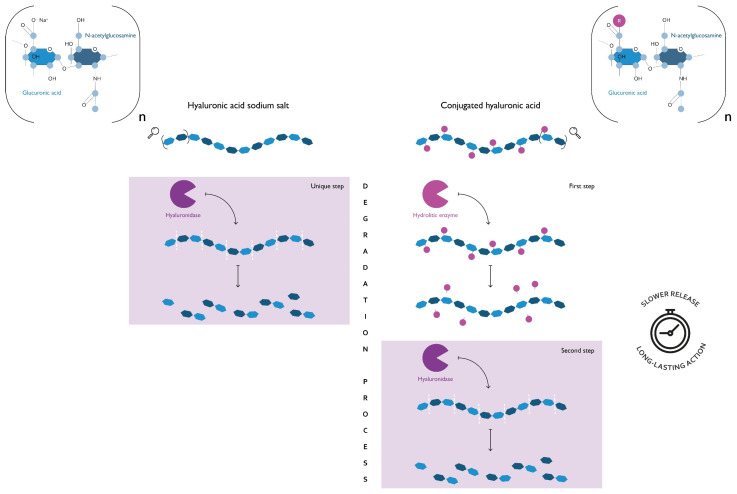
A comparison of the degradation process of hyaluronan and conjugated hyaluronic acid (HA). Both polymers are composed of a variable number of dimers (n dimers) of glucuronic acid and N-acetylglucosamine, linked via alternating β-1,4 and β-1,3 glycosidic bonds. Hyaluronan is the sodium salt of hyaluronic acid (HA) and represents the form that naturally occurs in the human body. The conjugated HA is obtained through the introduction of chemical modifications of the HA backbone. As can be seen from this schematic representation, the presence of a covalently bonded functional group (R) has an impact on the polymer degradation process. If on one hand, the β-1,4 glycosidic bonds of hyaluronan are easily accessible for hyaluronidase, on the other, those of conjugated HA to be such require a preliminary step where an additional hydrolytic enzyme removes the functional group. This difference implies that the degradation products of conjugated HA are released over an extended period, thus ensuring a long-lasting action of the products that contain it. Vaginal moisturizers containing conjugated HA and, therefore, having a prolonged residence time on the mucosa, have been described by Cascone & Lamberti (2019) [85].

HA is ubiquitously expressed in the human body, but the highest amounts are found in the ECM of soft connective tissues; it is particularly abundant in the skin and in ECM-rich districts such as the synovial joint fluid, the vitreous body of the eyes, and the umbilical cord. It has a fast turnover: the balance between synthesis and degradation of HA plays a key role in the regulation of its activity, controlling not only the amount but also the molecular weight of HA. In fact, high molecular weight (HMW) and low molecular weight (LMW) HA can exert different and even opposite biological effects acting through two distinct mechanisms: (i) as a passive structural molecule, due to its physicochemical properties; (ii) as an active signaling molecule, through interaction with proteoglycans or cell surface receptors (Figure 1) [12,84]. In particular, vaginal moisturizers containing conjugated HA have a prolonged residence time on the mucosa [85].

HA is involved in several physiological processes, including tissue homeostasis and regeneration, wound healing, inflammation, cell migration and proliferation, and embryonic development [12]. HA plays a relevant role also in the urogenital system as, under physiological conditions, it helps to maintain water balance and tissue integrity. 

Due to its ubiquitous expression in the human body, its peculiar biological and physicochemical features, and its high safety profile, HA has been widely used for various medical applications [86] apart from GSM. It is employed in dermatology for the formulation of skincare and wound-healing products [87]. In ophthalmology, it is applied both in surgery and as ophthalmic solution for the relief of dry eye [82,88]. It can be favorable in otology operations [89]. Moreover, it can be useful in the treatment of patients with osteoarthritis [90], asthma [91], cystitis [92,93], and vesicoureteral reflux [94].

## 7. Hyaluronic Acid as a Valid Non-Hormonal Option for GSM

As mentioned above, HA is naturally present in several body parts [95], including the urinary and genital tissues, where it maintains appropriate levels of hydration thereby promoting tissue elasticity and alleviating vaginal dryness. Vaginal moisturizers based on HA and HA-derived polymers have shown interesting clinical results in the treatment of GSM-associated symptoms, both in terms of efficacy and safety/tolerability [15]. Indeed, HA not only has a protective effect on the vaginal mucosa, but LMW HA molecules are also able to penetrate into the deeper layers of the vaginal epithelium [96]. To improve polymer stability, hydration degree, and other biological properties, several HA derivative biopolymers have been created through esterification [97], and many different formulations are available for intravaginal administration, including gels, creams, douches, tablets, and suppositories. Table 1 reports an overview of studies available in clinicaltrial.gov concerning different HA formulations for the treatment of dryness and other symptoms associated with GSM. At present, only a small number of clinical studies have been published in cancer survivors, whereas more data are available on the efficacy of HA for the relief of urogenital symptoms and sexual dysfunction in non-oncologic samples [98,99,100,101,102,103].

De Seta et al. [104], for example, demonstrated the efficacy, tolerability, and safety of 12-week treatment with a new vaginal gel containing HA along with sea buckthorn (Hippophaë rhamnoides) oil, aloe vera, 18β-glycyrrhetic acid, and glycogen in 60 postmenopausal women with symptoms associated with GSM. Indeed, the gel was effective in reducing vaginal pain, dyspareunia and vaginal pH, with the Vaginal Health Index showing significant improvement at day 90 (*p <* 0.0001), and in reducing each GSM symptom (vaginal dryness, vaginal itching, burning sensation) at weeks 2 and 4, and the end of the study (*p <* 0.0001). The analysis of self-reported Female Sexual Function Index scores showed, after the end of treatment, an improvement of sexual function in the active-treatment group, with a statistically significant increase (*p <* 0.001) in all domains scores and total score (*p <* 0.001). Almost all subjects in the active group reported excellent tolerability of vaginal gel and declared to be happy or very happy for: satisfaction with current treatment, convenience, improvement of their medical condition, rating compared to their previous treatment, the recommendation to others and continuation of their treatment. The assessment of AEs and data collected by laboratory test, physical examination and vital signs confirmed the safety of the tested product. An open-label, uncontrolled prospective study on 150 postmenopausal women showed that vaginal suppositories containing HA, vitamin E and vitamin A significantly improved vaginal dryness (from 7.92 at baseline to 4.22 at visit 1, to 0.84 at visit 2, and to 0.0 at visit 3 (28 days, end of the treatment)) as well as other signs and symptoms associated with GSM such as itching (from 3.82 at baseline, to 2.89 at visit 1, to 1.35 at visit 2, and to 1.03 at visit 3), burning (from 3.63 at baseline, to 2.45 at visit 1, to 1.31 at visit 2, and to 1.03 at visit 3), dyspareunia (from 2.88 at baseline, to 2.19 at visit 1, to 1.43 at visit 2, and to 1.04 at visit 3), and vaginal inflammation (from 2.71 at baseline, to 1.93 at visit 1, to 1.65 at visit 2, and to 1.04 at visit 3), swelling or irritation (from 2.45 at baseline, to 1.78 at visit 1, to 1.34 at visit 2, and to 1.00 at visit 3). The compliance to the treatment was complete for 126 patients and partial for the other 4 patients. The overall judgement of the product’s effectiveness reported by the investigator was optimal in 108 patients, good in 20 patients, and moderate in 2 patients. The overall judgement of the product’s safety was reported by the investigator optimal in 124 patients, good in 4, and sufficient in 2 patients. Acceptability of the product was evaluated by the patients as decidedly acceptable by 97 patients, easily acceptable by 31, and acceptable by 2 patients [105]. Consistently, a prospective, observational study involving 46 postmenopausal women demonstrated that the treatment with an HA-based preparation significantly reduced vaginal dryness, burning, itching, and dyspareunia. The treatment protocol consisted of the administration of a hyaluronic acid-based liquid preparation for vaginal use three times a week, for a total of 8 weeks. The Vaginal Health Index showed a statistically significant improvement from the value of 9.65 to 19.96. At the end of treatment, all the baseline VAS scores showed a statistically significant (*p* < 0.0001) improvement, and a 95% degree of patients’ satisfaction was obtained [96]. A number of randomized-controlled trials (RCTs) supported these encouraging results. A placebo controlled RCT involving 36 postmenopausal women reported a significant reduction in vaginal dryness in both HA and placebo groups applying a gel formulation daily but itching and burning were significantly improved only in the HA group. Both treatments were very well tolerated, and compliance of the treatment was very high [106]. HA-based pessaries efficacy was confirmed recently in a multicenter, non-controlled prospective study involving 40 postmenopausal women that showed a significant amelioration of GSM-associated vaginal signs and symptoms. The number of patients with an overall Vaginal Health Index score ≤15, the threshold value for GSM diagnosis, decreased from 100% at screening and baseline visits to 50% and 20% at the 4-week and 12-week follow-up visits, respectively. The mean total score for patient’s GSM symptom perception improved significantly (*p* < 0.0001), decreasing from 7.0 ± 2.45 at baseline to 2.5 ± 2.09 and 0.5 ± 0.85 after 1 and 3 months, respectively. Patient’s perception of vaginal dryness improved in at least one score in 97.5% of patients. Patients’ overall satisfaction was very high, as 25 patients (62.5%) were greatly satisfied, and 14 patients (35.0%) were satisfied to very satisfied with the treatment after 3 months. Thirty-seven patients (92.5%) positively appreciated study treatment already after 1 month. Excellent local tolerability was reported by all patients at both follow-up visits [101]. In all these studies, HA treatment resulted safe and very well tolerated, and overall patient satisfaction was high. 

Some RCTs were designed to compare the efficacy of HA-based preparations with that of topical hormone treatments. In a multicenter open-label RCT of 144 women, an HA-based vaginal gel and an estriol cream significantly relieved vaginal dryness and other vaginal symptoms, with no significant differences between the two arms (84% vs. 89%, respectively). The vaginal microenvironment remained unaffected by the treatment in 80.6% of subjects in the hyaluronic acid vaginal gel group and 77.27% in the estriol cream group. After 1 month of hyaluronic acid treatment, 56.96 ± 41.47% of the patients reported that pain during intercourse was reduced, which illustrated the effectiveness of the treatment. The treatment in both groups was applied every 3 days for a total of 10 applications: Hyaluronic acid vaginal gel was supplied in a 30 g aluminum tube with a vaginal applicator, which provides a dose of around 5 g. Estriol cream was supplied in a 15 g vial with a prefilled applicator, providing a dose of around 0.5 g [100]. In another RCT on 56 postmenopausal women, GSM signs and symptoms were significantly enhanced both by HA and estrogen treatments. Estrogen 0.625 mg cream and HA vaginal cream (containing 5 mg sodium salt) were applied for a period of 8 weeks (one applicator of drug (0.058 mg) every night before sleep for a period of two weeks and two times a week for the next six weeks and one applicator 5 mg every night before sleep for a period of 8 weeks, respectively). However, urinary incontinence improved only in the HA group, and the degree of improvement in dryness, maturation index and composite score of vaginal symptoms was greater in the HA group showing a reduction in the estrogen group at a rate of 1.7 ± 0.62 and in the hyaluronic group at a rate of 3.32 ± 0.76 [107]. On the other hand, a RCT comparing HA and estradiol treatment for 8 weeks (25 μg estradiol vaginal tablets daily for 14 days, and subsequently, one tablet twice per week or vaginal tablets containing hyaluronic acid sodium salt 5 mg once in a day) in 42 women showed that, even though GSM symptoms, vaginal pH, and vaginal maturation improved significantly in both groups, symptom relief (from 9.71 ± 1.93 to 2.67 ± 1.53 in the estradiol group, from 9.24 ± 1.92 to 3.86 ± 1.39 in the hyaluronic acid group) and maturation index (from 4.38 ± 0.80 to 71.19 ± 12.96 in the estradiol group, from 4.14 ± 0.85 to 44.40 ± 9.32 in the hyaluronic acid group) were significantly superior in the estradiol group [108]. Finally, an open-label RCT in 74 women showed that both HA vaginal gel and estrogen cream reduced patients’ perception of vaginal dryness symptoms to a similar extent during a 3 weeks treatment period (the percentage of participants who reported acceptance for attributes and perceived efficacy results evaluated after 21 +/− 2 days of use of products on the test was 97 ± 1 for HA and 100 for estrogen, respectively) [109].

Despite the relative low number of patients included in the studies and missing information on the dosage used for some of them, the clinical testing has demonstrated that intravaginal treatment with HA-based products successfully reduces GSM symptoms in postmenopausal woman, and their efficacy is superimposable to that of topical estrogen preparations at least in the short term. The beneficial effect of HA treatment was also evidenced by the high degree of patients’ satisfaction. Furthermore, its favorable safety profile and excellent local tolerability were confirmed.

There is a paucity of clinical studies currently available testing the activity of HA in cancer survivors suffering from GSM. In a randomized pilot trial, 57 postmenopausal women diagnosed with early-stage BC and in their first year of AI therapy received together with a vaginal lubricant and dilator, access to an educational website and phone coaching—either an HA-based moisturizer, a prebiotic-based moisturizer, or no additional therapy. After 6 months of treatment, the improvement of sexual function assessed by using the female sexual function index (FSFI) was significantly higher in the HA group as compared to the prebiotic group: mean score 25.90 and 15.08, respectively [100]. Although this was a pilot study needing further confirmation, its findings suggest that an early intervention with HA can mitigate the sexual problems caused by AI therapy, preventing or delaying the onset of symptoms [100]. Another pilot study recently confirmed the potential of HA-based moisturizers for the treatment of GSM in cancer survivors: 101 women either with a history of hormone receptor-positive (HR+) BC undergoing adjuvant therapy with an AI, or with HR+ endometrial cancer treated with surgery and postoperative radiotherapy, used an HA-based vaginal gel to relieve GSM-associated symptoms for up to 24 weeks. Self-perceived GSM symptoms, FSFI scores, vaginal pH, and the menopausal symptom checklist (MSCL) score improved significantly at the end of the treatment compared to baseline, highlighting that HA-based moisturization was able to improve vulvovaginal health and sexual function in these patients. Indeed, seventy-two percent (*n* = 56/78) of the patients indicated improvements in vaginal symptoms and 21% (*n* = 16/78) indicated a partial benefit. Ninety-two percent of all patients (72/78 patients with vaginal symptoms and 46/50 patients with vulvar symptoms) attributed the improvement to the HLA-based treatment or felt the treatment was partly responsible for symptom relief. By study completion, 94% (61/65) of the women felt their vaginal symptoms and 91% (41/45) felt their vulvar symptoms had been somewhat to very resolved. Ninety-eight percent (63/64) indicated they would recommend the HLA-based product to another female cancer survivor [110].

Therefore, HA treatment seems effective in relieving GSM symptoms also in cancer patients or survivors. Together with its safety and tolerability, this makes HA-based formulations good candidates for use in the oncological population.

## 8. Conclusions

Experimental evidence suggests that HA-based moisturizers are effective, safe, and well tolerated and may represent a valid option for the management of GSM in patients who are unable or unwilling to undergo hormone-based therapies, such as women with a history of cancer. In BC survivors, HA depletion that takes places in several body parts (including urogenital tissues) may be worsened by the adjuvant therapy with AIs and SERMs, thus potentially accelerating the development of GSM-associated symptoms. HA replenishment strategies could thus be integrated early into GSM treatment protocols for BC patients. 

A joint effort should be made by oncologists, gynecologists, and all HCPs to help women overcome the psychological and cultural barriers that make them reluctant to address issues concerning their intimate life. Early recognition and treatment of GSM symptoms could prevent or delay their worsening, allowing women who have won their battle against cancer not just to survive, but fully enjoy life.

## Figures and Tables

**Table 1:** Hyaluronic acid (HA)-based vaginal products available for the treatment of GSM.

Product Name	Main Components	Formulation	Producer	Indications	Clinical Trials
HYALO GYN^®^ HYALOFEMME^®^	Prolonged-release hyaluronic acid derivative (Hydeal-D^®^)	Gel, suppository	Fidia Farmaceutici (Abano Terme, Padova, Italy)	Treatment of dryness of various origin; aid in the natural healing process of friction-induced microlesions in the vaginal mucosa	NCT04355403-Performance and Safety of Hyalo Gyn Gel on the Treatment of Vaginal Atrophy in Postmenopausal Women [98]
					NCT01603303-Preventing Sexual Dysfunction in Women on Aromatase Inhibitors [99]
					NCT01557179-Evaluation of the Efficacy and Safety of Hyaluronic Acid Vaginal Gel to Ease Vaginal Dryness [100]
					NCT03557398-Efficacy and Safety of HYDEAL-D Vaginal Pessaries Application on the Treatment of Vaginal Atrophy in Post-menopause Women [101]
					NCT04560283-HYDEAL-D^®^ Application for Promoting the Restoration of Sexual Function in the Postpartum Period (HYDEAL-D) [102]
REVAREE^®^	Hyaluronic acid sodium salt	Suppository	Bonafide (Milton, Canada)	Dryness or discomfort, vaginal atrophy	NCT04544475-A Randomized, Single Center Pilot Study Comparing Hyaluronic Acid to Vaginal Estrogen for Treatment of Genitourinary Syndrome of Menopause
MUCOGENE^®^	Hyaluronic acid with liposomal structure	Gel, ovule	Iprad (Gentilly, France)	Hydration, lubrication and healing of mucous membrane	NCT04664985-Evaluation of Mucogyne^®^ Ovule in Vulvovaginal Dryness Management in Women Treated by Brachytherapy and/or Radiotherapy for Endometrial or Cervical Cancer
					NCT04713917-Evaluation of Innovative Therapeutic Approaches of Vaginal and Sexual Dysfunction After Breast Cancer Treatment
CICATRIDINA^®^	Hyaluronic acid plus herbal extracts	Suppository	Farma-Derma (Sala Bolognese, Italy), Angelini (Wien, Austria)	Adjuvant treatment of reparative processes in atrophic and dystrophic conditions of the mucosa	NCT03816735-Laser vs. Hyaluronic Acid for GSM in Breast Cancer
GYNOMUNAL^®^	Hop extract, Vitamin E, hyaluronic acid	Gel	Cederberg (Binningen, Switzerland)	Dryness or discomfort, vaginal atrophy	NCT01948583-Humectant Activity of a New Formulation of Gynomunal^®^ Vaginal gel
					NCT02269826-Efficacy and Safety of Non-hormonal Vaginal Preparations in Treating Vaginal Dryness [103]

## Data Availability

Not applicable.
